# Whole-Genome Sequence Analysis and Probiotic Characterization of 5-Methoxytryptophan-Producing Strain *Lacticaseibacillus paracasei* RM081

**DOI:** 10.3390/microorganisms14071431

**Published:** 2026-06-30

**Authors:** Yu-Yi Chen, Alican Abay, Muhammet Ali Asan, Yu-Chun Lin, Yen-Po Chen

**Affiliations:** 1 Department of Animal Science, National Chung Hsing University, Taichung City 402, Taiwan; 2 The iEGG and Animal Biotechnology Research Center, National Chung Hsing University, Taichung City 402, Taiwan; 3 Department of Biotechnology and Animal Science, National Ilan University, Yilan City 260, Taiwan

**Keywords:** probiotics, *Lacticaseibacillus paracasei*, whole-genome sequence, 5-methoxytryptophan, anti-inflammation

## Abstract

This study comprehensively examines the whole-genome sequence and probiotic potential of *Lacticaseibacillus paracasei* RM081, a strain originally isolated from raw bovine milk. Whole-genome sequencing and in silico analyses provided a robust molecular basis for its functional traits. The *L. paracasei* RM081 genome harbors an extensive repertoire of carbohydrate-active enzymes, suggesting strong prebiotic utilization capabilities. Crucially, genomic mining identified key genetic determinants for postbiotic synthesis, including the potential to synthesize the anti-inflammatory metabolite 5-methoxytryptophan (5-MTP). Moreover, comprehensive safety evaluations confirmed the absence of transferable antimicrobial resistance genes, virulence factors, biogenic amine-producing genes, and plasmids, indicating a secure genomic architecture without horizontal gene transfer risks. These genomic predictions were further substantiated by valid in vitro phenotypic models. The strain exhibited strong tolerance to gastric acid, maintaining high viability at pH 3.5 and 2.5 after 4 h, and survived well at 0.1% bile salt concentration. Furthermore, *L. paracasei* RM081 demonstrated robust cell surface properties, with a high auto-aggregation rate (85.0 ± 0.7%), hydrophobicity (71.5 ± 2.4%), and 78.0 ± 4.8% adhesion to Caco-2 intestinal epithelial cells, supporting its potential for colonization. Regarding antioxidant capacity, the cell-free supernatant displayed the highest DPPH scavenging activity (37%), indicating the active secretion of antioxidative metabolites. Collectively, these findings establish *L. paracasei* RM081 as a highly promising, safe probiotic and postbiotic candidate with verified colonization potential and functional capabilities.

## 1. Introduction

Probiotics are defined as “live microorganisms that, when administered in adequate amounts, confer health benefits to the host” [1]. To effectively deliver these benefits, successful gut colonization and survival under gastrointestinal conditions are crucial. Lactic acid bacteria (LAB), particularly the *Lactobacillaceae* family, are widely recognized for their ability to colonize the intestinal tract, improve microbiota balance, and reinforce gut barrier functions [2,3,4]. Taxonomically, lactic acid bacteria (LAB) are Gram-positive, non-spore-forming, catalase-negative cocci or rods belonging to the order *Lactobacillales* (phylum *Bacillota*). Biochemically and metabolically, they are acid-tolerant bacteria characterized by their ability to ferment carbohydrates, producing lactic acid as the primary metabolic end-product. The establishment of these bacteria in the gut allows them to directly interact with host tissues and employ diverse mechanisms to prevent pathogen colonization. These host protection mechanisms include the production of antimicrobial metabolites (e.g., lactic acid, organic acids that lower pH, hydrogen peroxide, and bacteriocins), the competitive exclusion of pathogens by blocking mucosal binding sites (mediated by sortase-anchored proteins, enolases, and glyceraldehyde-3-phosphate dehydrogenase), and immunomodulatory effects, such as reinforcing intestinal tight junctions and stimulating secretory IgA (sIgA) production [5,6,7]. Among these, *Lacticaseibacillus* species have garnered significant attention. Well-characterized probiotic strains, such as *L. rhamnosus* GG and *L. casei* Shirota, are widely documented to enhance gut integrity, modulate immune cells, and inhibit pathogens. *L. paracasei* in particular has been designated “Qualified Presumption of Safety” (QPS) by the European Food Safety Authority (EFSA) and is generally recognized as safe (GRAS) by the U.S. Food and Drug Administration (FDA). However, because the functional properties and safety profiles of probiotics are highly strain-specific, rigorous genomic and phenotypic safety evaluations of newly isolated strains remain indispensable before their application.

Beyond the established benefits of live colonization, recent studies have emphasized the therapeutic role of postbiotics and bioactive microbial metabolites produced by specific LAB strains [8,9,10,11,12]. Notably, a novel strain, *L. paracasei* RM081, which was isolated from bovine raw milk, shows that it could produce a unique anti-inflammatory compound, 5-methoxytryptophan (5-MTP), and further elicited a decrease in pro-inflammatory cytokine production in the RAW264.7 macrophage cell line upon lipopolysaccharide stimulation and amelioration of dextran sodium sulfate (DSS)-induced colitis in a mouse model [13]. 5-MTP exerts its protective effects by inhibiting cyclooxygenase-2 (COX-2) expression and NF-κB activation, thereby suppressing the release of pro-inflammatory cytokines and protecting endothelial and mucosal barriers [14]. The capacity to synthesize and secrete 5-MTP represents a highly specialized metabolic niche that distinguishes *L. paracasei* RM081 from traditional probiotic strains, which primarily rely on short-chain fatty acid (SCFA) production for anti-inflammatory effects. Interestingly, the genetic capacity for 5-MTP biosynthesis is not ubiquitous among LAB. Cross-species genomic comparisons and database mining indicate that the essential biosynthesis genes are restricted to specific lineages within the *L. casei* group and are conspicuously absent in other widely studied probiotic taxa, such as *L. rhamnosus*, *Lactiplantibacillus plantarum*, *Lactobacillus acidophilus*, *Limosilactobacillus reuteri*, and *Bifidobacterium* species. The innate capacity to synthesize 5-MTP significantly enhances the functional profile of *L. paracasei*, positioning it as a promising candidate for targeted therapies aimed at mitigating intestinal inflammation and maintaining mucosal homeostasis.

Despite these promising phenotypic traits, elucidating the underlying molecular mechanisms and ensuring the safety of novel probiotic strains are essential for their application. Whole-genome sequencing (WGS) serves as a vital tool to decode the genetic determinants responsible for postbiotic biosynthesis, adherence capabilities, stress resistance, and overall safety [15]. Therefore, in this study, a combination approach integrating genomic analysis and in vitro tests was used to comprehensively assess the safety and probiotic characteristics of *L. paracasei* RM081. These integrated assessments included WGS to precisely identify genetic markers associated with antimicrobial resistance, virulence factors, and biogenic amine synthesis, alongside phenotypic evaluations of its tolerance to gastrointestinal stressors, adhesion to intestinal epithelial cells, and antioxidant capacity. This combined approach provides a robust framework for establishing *L. paracasei* RM081 as a highly functional and safe probiotic candidate for therapeutic and nutritional applications.

## 2. Materials and Methods

### 2.1. Bacterial Strain and Growth Conditions

*Lacticaseibacillus paracasei* RM081 was isolated from raw bovine milk collected from the dairy research farm at National Chung Hsing University (Taiwan). The taxonomic identification of strain RM081 was performed in 2016 by the Bioresource Collection and Research Center (BCRC, Taiwan). Briefly, the 16S rRNA gene was amplified using the universal primers 27F (5′-AGAGTTTGATCCTGGCTCAG-3′) and 1492R (5′-GGTTACCTTGTTACGACTT-3′), followed by Sanger sequencing and BLAST (version 2.4.0) alignment against the NCBI GenBank database for identification [16]. The taxonomic assignment was verified through BLAST analysis against the NCBI database. The carbohydrate fermentation profile was determined using the API 50 CH system (bioMérieux, Marcy-l’Étoile, France). Cultures were routinely propagated in de Man, Rogosa, and Sharpe (MRS) broth (Difco, Becton Dickinson, Franklin Lakes, NJ, USA) at 37 °C.

### 2.2. Genomic DNA Extraction and Sequencing

Genomic DNA was extracted using the phenol-chloroform method and purified with RNase A treatment. High-molecular-weight genomic DNA (100 ng) was randomly fragmented to <500 bp using a Covaris S220 sonicator (Covaris, Woburn, MA, USA). The fragmented DNA was end-repaired, 5’-phosphorylated, and dA-tailed in a single reaction using the End Prep Enzyme Mix, followed by ligation of P5 and P7 adapters. Size selection was performed using AMPure XP beads to recover fragments of ~470 bp (insert size of ~350 bp). The adapter-ligated library was amplified via 8 PCR cycles, validated on an Agilent 2100 Bioanalyzer (Agilent Technologies, Santa Clara, CA, USA), and quantified with a Qubit 3.0 Fluorometer (Thermo Fisher Scientific, Waltham, MA, USA). The multiplexed libraries were sequenced on the Illumina NovaSeq 6000 platform (Illumina, San Diego, CA, USA) using a 2 × 150 bp paired-end (PE) configuration by a commercial provider (GENEWIZ Suzhou, China). For PacBio sequencing, genomic DNA was sheared, and 10 Kb double-stranded DNA fragments were selected. The DNA fragments were end-repaired and ligated with universal hairpin adapters following the manufacturer’s protocol to construct a SMRTbell library. Sequencing was conducted on the PacBio Sequel SMRT platform (Pacific Biosciences, Menlo Park, CA, USA).

### 2.3. Sequence Quality Control and Hybrid Assembly

Sequence quality assessment of the Illumina short reads was performed using FastQC (v0.11.9), followed by trimming of adapters and low-quality bases using Trimmomatic (v0.39). For PacBio long reads, quality control was conducted using SMRT Link (v10.1). A hybrid assembly approach was employed to obtain the complete genome sequence. The PacBio long reads were assembled de novo using HGAP4 and Falcon of WGS-Assembler (v8.2), and subsequently polished with PacBio reads (Quiver v2.3.3) and Illumina short reads (Pilon v1.23) to correct base errors.

### 2.4. Taxonomic Identification and Phylogenomics

A phylogenomic tree based on core-genome sequences was constructed using the Bacterial and Viral Bioinformatics Resource Center (BV-BRC, https://www.bv-brc.org/, accessed on 11 May 2026) [17] platform. The average nucleotide identity (ANI) values between the genome of *L. paracasei* RM081 and reference type strains were determined using the JSpeciesWeb Server (JSpeciesWS: https://jspecies.ribohost.com/jspeciesws/, accessed on 11 May 2026) [18], an online server for genome-based identification. Furthermore, digital DNA-DNA hybridization (dDDH) values were obtained using the Type (Strain) Genome Server (TYGS: https://tygs.dsmz.de/, accessed on 11 May 2026) [19] to confirm the species-level taxonomic assignment.

### 2.5. Genome Annotation and CAZyme Profiling

Gene prediction was conducted using Prodigal (v2.6.3). Non-coding RNAs, including rRNA and tRNA, were annotated using RNAmmer (v1.2) and tRNAscan-SE (v2.0), respectively. Functional annotations were carried out by aligning predicted protein sequences against multiple databases. Specifically, sequence alignments against the Non-Redundant (NR) and Carbohydrate-Active enZYmes (CAZy) databases were performed using DIAMOND BLASTp (v2.0.15) (E-value < 10^−3^), and against the Gene Ontology (GO) database using BLASTp/BLAST2go (v5.2) (E-value < 10^−3^). Alignments to the Kyoto Encyclopedia of Genes and Genomes (KEGG) and Clusters of Orthologous Groups (COG) databases were performed using BLASTn and rpstblastn, respectively, with stricter E-value cutoffs (<10^−5^). Annotations were assigned based on the best hits.

### 2.6. Comparative Genomics and Gene Mining

Genes associated with virulence factors (VFs) and toxins were evaluated in the *L. paracasei* RM081 genome by cross-referencing the Virulence Factor Database (VFDB) [20]. Antimicrobial resistance (AMR) genes were investigated using the ResFinder (https://genepi.food.dtu.dk/resfinder, accessed on 13 May 2026) [21] database according to its default settings. To detect mobile genetic elements, the PHAge Search Tool Enhanced Release (PHASTEST, https://phastest.ca/, accessed on 13 May 2026) [22] and PlasmidFinder (https://cge.food.dtu.dk/services/PlasmidFinder-2.0/, accessed on 13 May 2026) [23] databases were utilized. For comparative genomics, reference genomes were selected from the NCBI Assembly database based on the following criteria: (a) complete genome assembly level; (b) taxonomic assignment to *Lacticaseibacillus paracasei*; (c) high completeness (>85%) and low contamination (<12%) validated via CheckM; and (d) representation of diverse ecological niches. The general genomic characteristics of the 20 representative *L. paracasei* genomes used for comparative genomics are provided in Supplementary Table S1. Pan-genome analysis was performed using the BV-BRC platform, incorporating specific reference genomes, including *L. paracasei* strains JCM 8130 (RefSeq assembly accession GCF_000829035.1), 8700:2 (GCF_000155515.2), and Zhang (GCF_000019245.4). OrthoVenn3 [24] facilitated the analysis of genomic orthologous clustering. Furthermore, to investigate the genetic basis of 5-MTP biosynthesis, comparative genomic screening was conducted to detect the presence of key putative genes—specifically, antibiotic biosynthesis monooxygenase (PGF_02016021) and SAM-dependent methyltransferase (PGF_02017057)—across the *L. casei* group and distantly related probiotic species, including *Lacticaseibacillus rhamnosus* NCTC13764 (GCF_900636965.1), *Lactiplantibacillus plantarum* SRCM100442 (GCF_009913655.1), *Lactobacillus acidophilus* ATCC 4356 (GCF_034298135.1), *Lactobacillus delbrueckii* subsp. *bulgaricus* SCB0695 (GCF_035757545.1), *Lactobacillus helveticus* TCI357 (GCF_046109915.1), *Limosilactobacillus reuteri* subsp. *simiae* LR66 (GCF_020784875.1), *Bifidobacterium animalis* subsp. *lactis* DSM 10,140 (GCF_000022965.1), and *Bifidobacterium longum* subsp. *longum* JCM 1217 (GCF_000196555.1). To construct the phylogenetic tree of the 5-MTP biosynthesis genes, the nucleotide sequences of the monooxygenase and methyltransferase genes were retrieved from the respective genomes. Multiple sequence alignment was performed using MUSCLE. The phylogenetic tree was constructed using the Maximum Likelihood (ML) method in MEGA 11 software, with 1000 bootstrap replicates to assess node support. The resulting tree was visualized and annotated using the Interactive Tree Of Life (iTOL) platform.

### 2.7. Acid and Bile Salt Tolerance Assays

To evaluate acid tolerance, overnight cultures of *L. paracasei* RM081 were harvested, washed, and resuspended in MRS broth adjusted to pH 1.5, 2.5, 3.5, or 4.5 using 3 M HCl (Fluka, Sigma-Aldrich, St. Louis, MO, USA). After a 4 h incubation at 37 °C, viable cell counts were determined via plate counting on MRS agar. For bile salt tolerance, cultures were inoculated into MRS broth containing 0.1%, 0.3%, or 0.5% (*w*/*v*) bile salt (oxgall; Sigma-Aldrich, St. Louis, MO, USA) and incubated at 37 °C. Viable counts were assessed after 4 h of incubation. Survival was calculated by comparing the colony-forming units (CFU/mL) before and after the treatments.

### 2.8. Antioxidant Activity Assays

The antioxidant capacity of *L. paracasei* RM081 was determined using the 2,2-diphenyl-1-picrylhydrazyl (DPPH; Alfa Aesar, Ward Hill, MA, USA) radical scavenging assay [25]. The strain was cultured in MRS broth at 37 °C for 24 h, and three distinct bacterial preparations were prepared as follows:

#### 2.8.1. Live Cells

The bacterial pellet obtained from centrifugation was washed twice with sterile phosphate-buffered saline (PBS; 0.01 M, pH 7.4, Sigma-Aldrich, USA) and resuspended in PBS. The cell density was adjusted to an optical density at 600 nm (OD_600_) of 1.0 (approximately 10^9^ CFU/mL).

#### 2.8.2. Cell-Free Extract (CFE)

To obtain intracellular components, the washed and resuspended intact cell suspension was subjected to ultrasonic disruption using an ultrasonic processor (Qsonica, Newtown, CT, USA) at 70% amplitude for 20 min in an ice bath to prevent thermal degradation of enzymes. The lysed mixture was centrifuged at 5000× *g* (5000 rpm) for 15 min at 4 °C to remove intact cells and cellular debris, and the resulting supernatant was filtered through a 0.22-μm filter.

#### 2.8.3. Cell-Free Fermentation Supernatant (CFS)

The 24 h bacterial culture was centrifuged at 1700× *g* (1700 rpm) for 5 min at room temperature. The supernatant was collected and passed through a 0.22-μm pore size membrane filter (Millipore, Burlington, MA, USA) to ensure the complete removal of bacterial cells.

To perform the assay, 100 μL of each preparation (CFS, intact cells, or CFE) was mixed with 100 μL of freshly prepared 0.2 mM DPPH solution in absolute ethanol in a 96-well microplate. For the control, 100 μL of sterile PBS was mixed with 100 μL of the DPPH solution. To account for the sample’s background absorbance and turbidity, sample blanks were prepared by mixing 100 μL of each bacterial preparation with 100 μL of absolute ethanol without DPPH. The reaction mixtures were incubated in the dark at room temperature for 30 min. The decrease in absorbance was measured at 517 nm using a microplate reader (BioTek, Winooski, VT, USA). The DPPH radical scavenging activity (%) was calculated using the following equation:

(1) $$\mathrm{DPPH}\mathrm{scavenging}\mathrm{activity}\left(\%\right)=\left(1-\frac{A_{\mathrm{sample}}-A_{\mathrm{blank}}}{A_{\mathrm{control}}}\right)\times 100,$$

where *A*_sample_ represents the absorbance of the sample mixed with the DPPH solution, *A*_blank_ represents the absorbance of the sample mixed with absolute ethanol, and *A*_control_ represents the absorbance of the PBS control mixed with the DPPH solution. All assays were performed in triplicate, and results are expressed as means ± standard deviations.

### 2.9. Carbohydrate Fermentation Profile Assays

The carbohydrate utilization profile of RM081 was assessed using the API 50 CH system (bioMérieux, France). Inoculations were prepared according to the manufacturer’s instructions and incubated at 37 °C for 48 h. The fermentation of 49 different carbohydrates was determined based on color changes in the medium, which were scored as positive (+), weakly positive (w), or negative (-). The species identification based on the resulting biochemical profile was performed and interpreted using the apiweb™ database identification software (version 5.1, bioMérieux, France; https://apiweb.biomerieux.com, accessed on 13 May 2026).

### 2.10. Cell Surface Hydrophobicity and Auto-Aggregation

Cell surface hydrophobicity was determined using n-hexadecane as the hydrocarbon phase. *L. paracasei* RM081 was cultured in MRS broth at 37 °C for 18 h, harvested by centrifugation (1700 rpm, 7 min, room temperature), and washed twice with PBS (pH 7.4). The cell pellet was resuspended in PBS to an optical density (OD_600_) of 1.0. A 3 mL aliquot of this suspension was mixed with 1 mL of n-hexadecane (Sigma-Aldrich, USA) and vortexed for 3 min. After a 1 h phase separation at room temperature, the aqueous phase was carefully collected, and the OD_600_ was measured. Hydrophobicity was expressed as the percentage decrease in the initial optical density of the aqueous phase.

Auto-aggregation ability was evaluated by adjusting the bacterial PBS suspension to an OD_600_ of 1.0 and incubating it at 37 °C without agitation. The OD_600_ of the upper phase was measured hourly over a 5 h period. The auto-aggregation percentage was calculated based on the reduction in optical density relative to the initial value. All experiments were performed in triplicate.

### 2.11. Adhesion to Intestinal Epithelial Cells, Caco-2

The adhesion ability of *L. paracasei* RM081 to human intestinal epithelial cells was evaluated using the Caco-2 cell line. Caco-2 cells were cultured in DMEM (Gibco, Grand Island, NY, USA) supplemented with 10% heat-inactivated FBS (Gibco), 1% non-essential amino acids, 1% L-glutamine, 20 μg/mL penicillin, and 20 μg/mL streptomycin. Cells were seeded at 1 × 10^5^ cells/well in a 24-well plate and grown to confluence at 37 °C in a 5% CO_2_ atmosphere. *L. paracasei* RM081 was grown for 18 h in MRS broth, harvested (3000 rpm, 7 min), washed with PBS, and resuspended at 1 × 10^8^ CFU/mL in DMEM. After washing the Caco-2 monolayers with PBS, 1 mL of the bacterial suspension was added per well and co-incubated for 3 h at 37 °C (5% CO_2_). Non-adherent bacteria were removed by washing twice with PBS. Caco-2 cells were then detached using 0.1% trypsin, resuspended in PBS, and the adhered bacteria were quantified by plating serial dilutions on MRS agar. The adhesion efficiency was calculated as the percentage of adhered bacteria relative to the initial population. For graphical representation of the relative adhesion ratio, the initial inoculated bacterial count was designated as the control group and normalized to 100%.

### 2.12. Antibiotic Susceptibility Tests

Antibiotic susceptibility was assessed via the agar disc diffusion method on MRS agar. The antibiotics tested included penicillin (10 units, Sensi-Disc, Becton Dickinson, Franklin Lakes, NJ, USA), erythromycin (15 μg, Sensi-Disc, USA), chloramphenicol (30 μg, Sensi-Disc), tetracycline (30 μg, Sensi-Disc), streptomycin (10 μg, Sensi-Disc), and novobiocin (5 μg, Sensi-Disc). Zones of inhibition were measured after a 24 h incubation at 37 °C, and the results were interpreted according to Clinical and Laboratory Standards Institute (CLSI) guidelines [26].

### 2.13. Statistical Analysis

All experimental data were analyzed using Student’s *t*-test or one-way ANOVA followed by Duncan’s multiple range test via SAS software (version 9.4, SAS Institute Inc., Cary, NC, USA). Values are expressed as the mean ± standard deviation (SD), and a *p*-value of <0.05 was considered statistically significant.

## 3. Results

### 3.1. Genome Assembly and General Features

**Figure 1:**
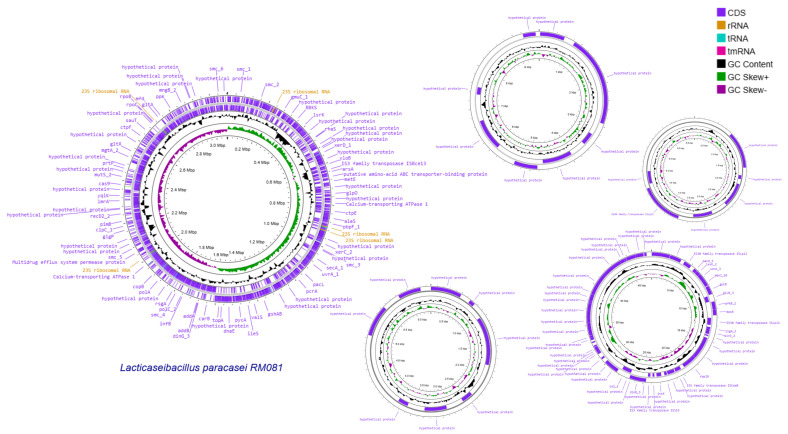
Circular representation of the *Lacticaseibacillus paracasei* RM081 genome map. Circles from outside to inside indicate: the first and second circles represent forward and reverse CDSs (coding sequences) annotated using Prokka, respectively, including tRNA, rRNA, and tmRNA; the third circle indicates the GC content; the fourth circle depicts the GC skew (G − C)/(G + C); and the fifth circle represents the genome size. The genome of *L. paracasei* RM081 comprises five contigs: a circular chromosome of 3,084,987 bp, and four plasmids (plasmid 1: 6696 bp, plasmid 2: 9600 bp, plasmid 3: 6373 bp, and plasmid 4: 47,991 bp).

The complete genome sequence of *Lacticaseibacillus paracasei* RM081 was successfully obtained using a hybrid assembly approach combining PacBio long-read and Illumina short-read sequencing (Figure 1). The assembly resolved into a circular chromosome of 3,084,987 bp (GC content of 46.26%) and four circular plasmids: Plasmid 1 (6696 bp, GC content of 41.52%), Plasmid 2 (9600 bp, GC content of 40.89%), Plasmid 3 (6373 bp, GC content of 42.11%), and Plasmid 4 (47,991 bp, GC content of 40.16%). The general genomic characteristics of *L. paracasei* RM081 are summarized in Table 1. The genome has a total size of 3.16 Mb, an average GC content of 46.19%, and a high N50 value matching the chromosome size (3.08 Mb), reflecting high assembly continuity.

Gene prediction identified 3096 protein-coding genes (CDSs), 59 tRNA genes, and 15 rRNA genes (representing 5 operons). Functional annotation of the CDSs was performed against multiple databases (Figure S1).

KEGG pathway mapping revealed that a significant proportion of genes were involved in carbohydrate metabolism, amino acid biosynthesis, membrane transport, and environmental adaptation. Genes related to lactic acid fermentation, stress response (including heat shock proteins and universal stress proteins), and short-chain fatty acid (SCFA) metabolism were also identified. These findings align with the strain’s phenotypic resilience under stress and its robust fermentative capabilities. COG classification showed that the most represented functional categories included “Carbohydrate transport and metabolism” (G), “Translation, ribosomal structure and biogenesis” (J), and “Replication, recombination and repair” (L), highlighting the strain’s adaptability and genetic stability.

CAZy database analysis further elucidated the carbohydrate-active enzyme profile of L. paracasei RM081. The genome harbors a diverse repertoire of carbohydrate-active enzymes, including various glycoside hydrolases (GHs) and glycosyl transferases (GTs), which play essential roles in polysaccharide degradation and utilization. This rich genomic capacity strongly supports the wide carbohydrate fermentation profile observed in the API 50 CH assay, indicating the strain’s potential for dietary fiber adaptation and prebiotic oligosaccharide metabolism.

Among the plasmids, Plasmid 1 (6.7 Kb), Plasmid 2 (9.6 Kb), and Plasmid 3 (6.4 Kb) are small cryptic plasmids primarily carrying plasmid replication proteins (RepB family, e.g., 2_2, 3_6, 4_3) and partition proteins, alongside hypothetical proteins. Plasmid 2 also encodes a putative peptidoglycan-binding domain protein (3_2) involved in cell-wall anchoring. In contrast, Plasmid 4 (48 Kb) is a larger, mobilizable/conjugative plasmid containing 56 genes. Notably, Plasmid 4 carries a complete mannose/fructose phosphotransferase system (PTS) sugar transporter operon IIABCD (5_4 to 5_7), its transcriptional regulator MurR/RpiR (5_9), and 1-phosphofructokinase (5_10), indicating its involvement in carbohydrate fermentation. Furthermore, Plasmid 4 harbors a type II restriction-modification system consisting of an LlaJI family restriction endonuclease (5_16) and a modification methylase (5_18) that serve as a defense system against foreign DNA. Conjugation-related elements, including the type IV secretion system protein VirD4 (5_45), the PcfB family transfer protein (5_44), and the plasmid mobilization relaxosome protein MobC (5_56), are also present on Plasmid 4.

### 3.2. Taxonomic Assignment and Phylogenomic Analysis

**Figure 2:**
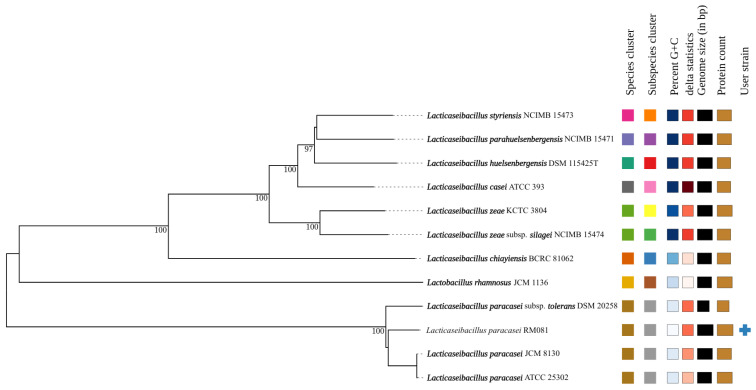
Core-genome phylogenomic tree of *Lacticaseibacillus paracasei* RM081 and related reference strains. The tree was inferred using the Type Strain Genome Server (TYGS) pipeline. The color panels display key genomic characteristics and taxonomic boundaries: Species cluster (demarcated by a 70% dDDH threshold) and Subspecies cluster (demarcated by a 79% dDDH threshold), where identical colors indicate membership within the same species or subspecies. Additional columns represent Percent G + C, delta statistics, Genome size (in bp), and Protein count (the width of the bars corresponds to the respective numerical values). The blue plus sign (+) indicates the study strain (*L. paracasei* RM081).

To precisely determine the taxonomic status of *L. paracasei* RM081, phylogenomic analysis based on core genomes was performed. The average nucleotide identity (ANI) value between *L. paracasei* RM081 and the reference type strain *L. paracasei* JCM 8130 (GCF_000829035.1) was 97.91%. Core-genome-based digital DNA-DNA hybridization (dDDH) values obtained via TYGS confidently clustered *L. paracasei* RM081 within the *L. paracasei* species group (Figure 2). Because the ANI and dDDH values significantly exceed the established species delineation thresholds of 95–96% and 70%, respectively, the strain was conclusively assigned as *Lacticaseibacillus paracasei*.

### 3.3. Phenotypic Probiotic Properties

#### 3.3.1. Acid and Bile Salt Tolerance

**Figure 3:**
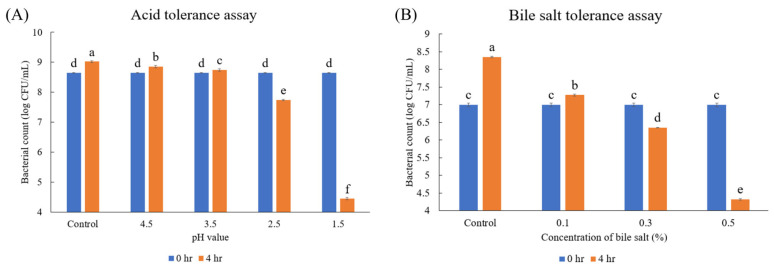
Survival of *Lacticaseibacillus paracasei* RM081 under simulated gastrointestinal conditions. (**A**) Acid tolerance assessed by measuring viable cell counts (CFU/mL) after 4 h of incubation at pH 1.5, 2.5, 3.5, and 4.5. (**B**) Bile salt tolerance evaluated after 4 h of exposure to 0.1%, 0.3%, and 0.5% (*w*/*v*) oxgall. Values are expressed as the mean ± standard deviation of triplicates. Different lowercase letters (a–f) above the bars indicate statistically significant differences (*p* < 0.05) among all groups within each panel, as determined by one-way ANOVA followed by Duncan’s multiple range test.

*L. paracasei* RM081 demonstrated resilience under simulated gastrointestinal conditions (Figure 3). In the acid tolerance assay (Figure 3A), the strain maintained high viability at pH 4.5 (7.27 × 10^8^ CFU/mL) and pH 3.5 (5.53 × 10^8^ CFU/mL) after a 4 h challenge, showing no significant reduction compared to the initial control (1.06 × 10^9^ CFU/mL). At pH 2.5, viability decreased by approximately 1.3 log units to 5.56 × 10^7^ CFU/mL. However, survival was severely reduced under extreme acidity at pH 1.5 (2.77 × 10^4^ CFU/mL).

For bile salt tolerance (Figure 3B), the strain was exposed to oxgall for 4 h. *L. paracasei* RM081 survived exposure to 0.1% bile salt (1.86 × 10^7^ CFU/mL). A dose-dependent reduction in viability was observed at higher concentrations, decreasing to 2.22 × 10^6^ CFU/mL at 0.3% and 2.10 × 10^4^ CFU/mL at 0.5% bile salt.

#### 3.3.2. Antioxidant Activity

**Figure 4:**
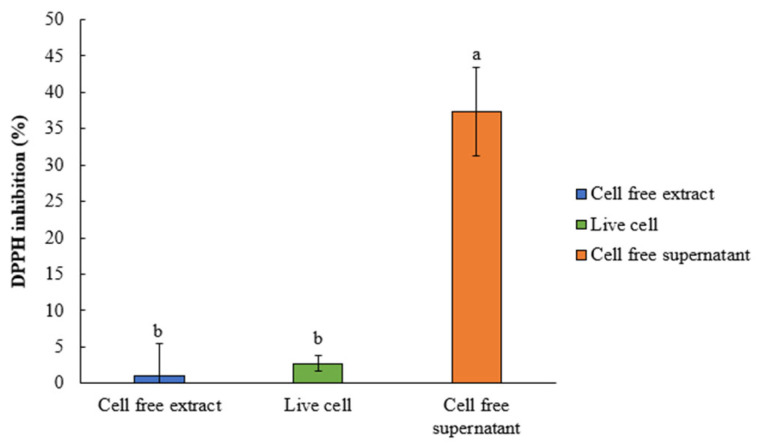
Antioxidant activity of *Lacticaseibacillus paracasei* RM081. DPPH free radical scavenging capacity (%) evaluated using intact live cells, cell-free extract (CFE), and cell-free supernatant (CFS). Means in the same figure with different letters differ significantly (*p* < 0.05) as determined by Duncan’s multiple range test. The error bars represent the SD (n = 3).

The DPPH radical scavenging activity of *L. paracasei* RM081 preparations is shown in Figure 4. The cell-free supernatant (CFS) exhibited the highest antioxidant capacity, with a scavenging rate of 37.0%, which was significantly higher (*p* < 0.05) than that of intact cells (5.2%) and the cell-free extract (CFE) (1.0%).

#### 3.3.3. Carbohydrate Fermentation Profile

Based on API 50 CH analysis (Table 2), *L. paracasei* RM081 fermented a broad range of carbohydrates, including glucose, galactose, fructose, mannose, L-arabinose, D-ribose, D-mannitol, D-lactose, D-sucrose, D-maltose, D-trehalose, D-melezitose, D-raffinose, gentiobiose, D-turanose, and D-tagatose. Negative reactions were observed for glycerol, erythritol, D-arabinose, D-xylose, L-sorbose, rhamnose, dulcitol, inositol, inulin, and starch.

#### 3.3.4. Auto-Aggregation and Surface Hydrophobicity

The cell surface properties of *L. paracasei* RM081 are summarized in Table 3. The strain exhibited a high auto-aggregation rate of 85.0 ± 0.7% after 5 h of incubation. In the microbial adhesion to hydrocarbons assay with n-hexadecane, the cell surface hydrophobicity was determined to be 71.5 ± 2.4%.

#### 3.3.5. Adhesion to Caco-2 Human Epithelial Cells

**Figure 5:**
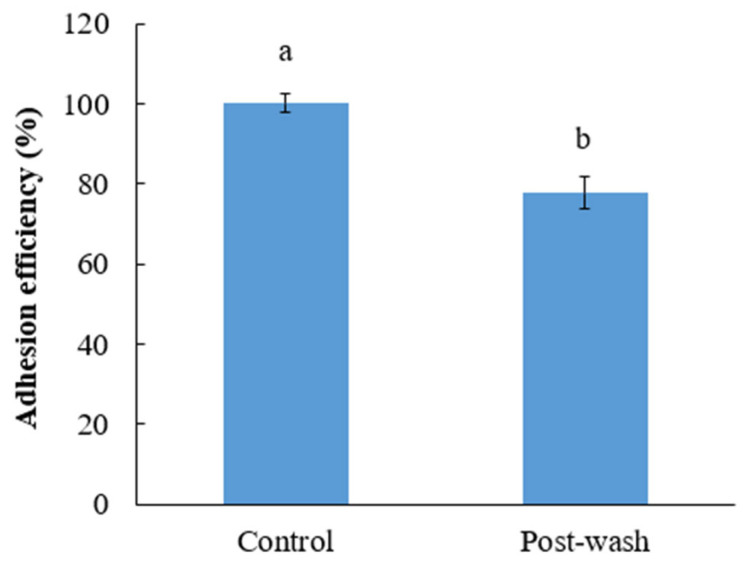
Relative adhesion ratio of *Lacticaseibacillus paracasei* RM081 to Caco-2 human intestinal epithelial cells. Control: Initial inoculated bacterial population (normalized to 100%); Post-wash: Adhered bacterial population remaining after phosphate-buffered saline (PBS) washing steps. Means in the same figure with different letters differ significantly (*p* < 0.05) as determined by Student’s *t*-test (*p* < 0.05). The error bars represent the SD (n = 3).

In vitro colonization potential was validated using the Caco-2 cell line model (Figure 5). *L. paracasei* RM081 exhibited a substantial adhesion rate of 78.0 ± 4.8% relative to the initial inoculum.

#### 3.3.6. Antibiotic Susceptibility Profile

The antibiotic susceptibility profile of *L. paracasei* RM081 is detailed in Table 4. The strain was susceptible to penicillin (35.0 ± 0.5 mm), erythromycin (32.0 ± 0.6 mm), chloramphenicol (30.0 ± 0.3 mm), and tetracycline (30.0 ± 0.3 mm). Conversely, it exhibited resistance to streptomycin (0 mm) and intermediate resistance to novobiocin (20.2 ± 0.4 mm).

### 3.4. Genotypic Characterization of Probiotic and Functional Traits

#### 3.4.1. Genomic Basis for Stress Tolerance

Genome annotation identified a complete F_0_F_1_-transporting ATP synthase operon (RM_1367 to RM_1374) mediating proton extrusion under acidic stress. While the classical bile salt hydrolase (*bsh*) gene was absent in the RM081 genome, alternative protection mechanisms were identified. These include major facilitator superfamily (MFS) and ATP-binding cassette (ABC) efflux pumps (*mdlA*/*smdA* at RM_2073, and *lmrB* at RM_2327) capable of active bile salt extrusion, as well as a capsular polysaccharide (CPS) biosynthesis gene cluster (RM_2203, RM_2204, RM_2711) that constructs a protective physical envelope barrier.

#### 3.4.2. Genomic Basis for Antioxidant Pathways

The genome of *L. paracasei* RM081 encodes a robust enzymatic and non-enzymatic reactive oxygen species (ROS) scavenging system. Intracellular antioxidant genes include NADH peroxidase (*npx* at RM_170, *npr* at RM_446), NADH oxidase (*nox2* at RM_254), superoxide dismutase (*sodA* at RM_2023), thiol peroxidase (*tpx* at RM_820), the thioredoxin system (*trxA* at RM_236, RM_575, RM_864 and *trxB* at RM_897, RM_1095), and glutathione reductase (*gor* at RM_670, RM_2326, RM_2791). Additionally, methionine sulfoxide reductases (*msrA* at RM_1407, RM_1584 and *msrB* at RM_1714) are present to repair oxidatively damaged proteins.

#### 3.4.3. Genomic Basis for Carbohydrate Utilization

The *L. paracasei* RM081 genome contains 252 genes belonging to Carbohydrate-Active enZYme (CAZyme) families. This carbohydrate utilization repertoire consists of 121 glycosyltransferases (GTs), 89 glycoside hydrolases (GHs), 14 carbohydrate esterases (CEs), 25 carbohydrate-binding modules (CBMs), 2 polysaccharide lyases (PLs), and 1 auxiliary activity (AA) enzyme, supporting the broad carbon fermentation spectrum shown in the API 50 CH assay.

#### 3.4.4. Genomic Basis for Cell Adhesion

Genomic mining revealed several sortase genes (class C sortase *srtA* at RM_494, RM_2166, RM_2167, RM_2285, RM_2555) that anchor LPxTG-containing surface proteins, WxL domain-containing proteins (RM_143, RM_679), and fibronectin-binding protein A (*fbpA* at RM_1635). This envelope-anchored machinery is complemented by genes encoding surface-exposed moonlighting proteins, including enolase (*eno* at RM_1115, RM_2628), glyceraldehyde-3-phosphate dehydrogenase (GAPDH, *gapA* at RM_1112, RM_1593), elongation factor Tu (*tuf* at RM_1524), and triosephosphate isomerase (*tpiA* at RM_1114). The exopolysaccharide (EPS) cluster (RM_985, RM_1179, RM_1180, RM_2203, RM_2204, RM_2711) supports surface aggregation and hydrophobicity.

#### 3.4.5. Intrinsic Susceptibility Markers

The phenotypic antibiotic susceptibility profile aligns with the intrinsic genetic markers identified in the genome. The resistance to streptomycin is mediated by intrinsic chromosomal alleles, specifically the ribosomal *rpsL* gene configuration. Intermediate resistance to novobiocin is similarly linked to the native DNA gyrase subunits (*gyrA* and *gyrB*). The absence of acquired resistance determinants underscores the intrinsic, non-transferable nature of these phenotypes.

### 3.5. Comparative Genomics and 5-MTP Biosynthesis Potential

**Figure 6:**
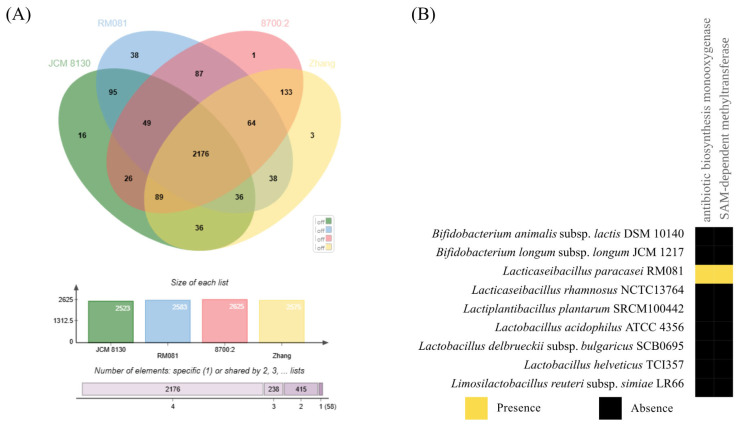
Comparative genomic analysis and 5-methoxytryptophan biosynthesis potential. (**A**) Venn diagram illustrating the pan-genome, core, and strain-unique genes among *Lacticaseibacillus paracasei* RM081, JCM 8130, 8700:2, and Zhang. A total of 2176 orthologous clusters were shared among all four strains, 238 were shared among three strains, and 415 were shared among two strains; 58 strain-specific singleton clusters were identified in RM081 (38), JCM 8130 (16), 8700:2 (1), and Zhang (3). (**B**) Distribution heatmap of specific genes associated with 5-methoxytryptophan (5-MTP) biosynthesis, demonstrating the presence of antibiotic biosynthesis monooxygenase (RM_1334) and SAM-dependent methyltransferase (RM_232) in the *L. casei* group and their absence in distantly related probiotic taxa. Yellow indicates the presence of the gene, while black indicates its absence.

Pan-genome comparison, visualized via a Venn diagram (Figure 6A), showed that *L. paracasei* RM081 shares a conserved core genome of 2176 orthologous clusters with reference strains JCM 8130, 8700:2, and Zhang, while harboring 58 strain-specific singleton genes.

Genome mining identified two chromosomal genes required for the biosynthesis of 5-methoxytryptophan (5-MTP): an antibiotic biosynthesis monooxygenase (PGF_02016021/1_1334) and a class I SAM-dependent methyltransferase (PGF_02017057/1_232). Heatmap analysis (Figure 6B) showed that while this two-gene pathway is conserved within the *L. casei* group (*L. paracasei* RM081, JCM 8130, 8700:2, Zhang), orthologs are absent in other commercial and reference probiotic strains (e.g., *L. rhamnosus*, *L. plantarum*, *L. acidophilus*, *L. delbrueckii*, *L. helveticus*, *L. reuteri*, *Bifidobacterium animalis*, and *B. longum*).

To evaluate the evolutionary origin of the 5-MTP pathway, the flanking genomic context and phylogenetic history were analyzed. In the RM081 chromosome, the methyltransferase (1_232) is located in a stable locus flanked by conserved metabolic genes, including an ABC transporter (1_231) and a threonine/serine exporter (1_237), and lacks any flanking transposases, integrases, or insertion sequences. The monooxygenase (1_1334) is flanked by a DNA/RNA endonuclease (1_1336) and a glycosyltransferase (1_1338). A phylogenetic tree of the 5-MTP monooxygenase and methyltransferase genes among the *L. casei* group and related bacteria reveals a vertical evolutionary topology that mirrors standard ribosomal species phylogeny (Figure S2), supporting vertical inheritance rather than horizontal gene transfer (HGT) as the primary mechanism.

### 3.6. Safety Evaluation

#### 3.6.1. Acquired Antibiotic Resistance Genes

Genome-wide screening using the ResFinder database detected no acquired, transferable antimicrobial resistance (AMR) genes in the chromosome or plasmids of *L. paracasei* RM081 (Table 5).

#### 3.6.2. Virulence Factors and Biogenic Amines

The Virulence Factor Database (VFDB) analysis confirmed the complete absence of true pathogenic toxins, enterotoxins, or hemolysins. Housekeeping genes that double as colonization determinants (putative adhesins *lap* and *efaA*, and moonlighting proteins *eno* and *gapA*) were detected, alongside capsule synthesis (*cps*) and two-component stress regulatory (*lisR*) genes. Stringent search criteria flagged a distant hemolysin transporter homolog, which is a native non-pathogenic membrane protein commonly found in LAB. Additionally, no genes associated with biogenic amine (BA) synthesis pathways (e.g., histidine decarboxylase, tyrosine decarboxylase, ornithine decarboxylase, lysine decarboxylase, agmatine deiminase) were present.

## 4. Discussion

### 4.1. Taxonomy of L. paracasei RM081

In this study, core-genome phylogenomics, ANI (97.91%), and dDDH values established *L. paracasei* RM081 as a member of the *L. paracasei* species group, aligning with species circumscription criteria [18]. The 3.16 Mb genome size and 46.19% GC content are consistent with other completed genomes of the species.

The genomic structure comprises a circular chromosome and four plasmids. Plasmid analysis reveals that Plasmids 1, 2, and 3 are small cryptic elements. Plasmid 2 encodes a peptidoglycan-binding domain protein that may contribute to cell envelope integrity and surface adhesion. In contrast, Plasmid 4 is a larger (48 Kb) plasmid containing a complete mannose/fructose PTS transport and fermentation operon. The presence of MurR/RpiR and 1-phosphofructokinase indicates that Plasmid 4 provides a metabolic niche, enabling the strain to utilize milk-derived sugars and adapt to raw bovine milk and intestinal environments. Plasmid 4 also carries an LlaJI type II restriction-modification system, which protects the host against bacteriophage infection and foreign DNA invasion, maintaining genomic stability. The presence of VirD4, PcfB-like, and MobC indicates that Plasmid 4 is a mobilizable plasmid; however, safety evaluation confirmed the complete absence of acquired AMR genes or toxins on this plasmid, ensuring that its transfer poses no risk to the gut microbiome.

### 4.2. Genotype-Phenotype Correlation in Probiotic Performance

*L. paracasei* RM081 exhibited high viability under simulated gastric acid (pH 3.5 and 2.5) and bile salt conditions. The genomic annotation explains this tolerance. Acid stress survival is mediated by the F_0_F_1_ ATPase operon, which actively pumps protons out of the cytoplasm at the expense of ATP [34]. Although the strain lacks a classical bile salt hydrolase (*bsh*), it utilizes a synergistic protection network to resist bile salt toxicity. This consists of ABC and MFS efflux pumps (such as *mdlA*/*smdA* and *lmrB*) that actively extrude bile salts from the cell, and the capsule polysaccharide (CPS) biosynthesis machinery that forms a physical envelope barrier [35].

The antioxidant assay showed that the cell-free supernatant (CFS) possesses the highest DPPH scavenging capacity (37%). Intracellularly, the strain utilizes enzymatic pathways (NADH oxidase, NADH peroxidase, superoxide dismutase, and thiol peroxidase) alongside the thioredoxin and glutathione systems to maintain intracellular redox balance and survive oxidative stress. However, these intracellular proteins cannot account for the extracellular antioxidant capacity. The high scavenging activity in the CFS is driven by secreted low-molecular-weight metabolites, organic acids, and the anti-inflammatory postbiotic 5-MTP, which is actively synthesized and secreted by the strain [10,13].

The ability of RM081 to ferment a broad range of carbohydrates, including prebiotic sugars (lactose, raffinose, melezitose, gentiobiose, turanose), correlates with its 252 CAZyme genes. The GH and GT repertoires allow the strain to break down and utilize complex plant-derived glycans and prebiotic fibers. This metabolic versatility allows the strain to adapt to diverse nutritional environments, enhancing its colonization potential in the human gut [36], and indicates RM081’s potential compatibility with plant-based or fiber-enriched food formulations, offering prebiotic synergy [37].

Surface hydrophobicity (71.5%) and auto-aggregation (85.0%) are key physical properties that facilitate cell adhesion. The strain showed high adhesion (78.0%) to Caco-2 intestinal epithelial cells. Genomically, this colonization potential is driven by several sortase genes that covalently anchor LPxTG-containing adhesion proteins to the cell wall peptidoglycan [38], WxL domain proteins, and fibronectin-binding protein A (fbpA) that directly mediates adherence to the host extracellular matrix [39]. Additionally, the presence of a glycosyltransferase and polysaccharide biosynthesis gene cluster involved in exopolysaccharide (EPS) production directly explains the cell envelope’s structural hydrophobicity and cell–cell auto-aggregation traits [40]. Furthermore, this envelope machinery is augmented by surface-exposed moonlighting proteins. Adhesion extends the residence time of the bacteria in the gut and promotes interaction with the host immune system [41]. Housekeeping proteins like Enolase (eno) and GAPDH (gapA) are known to bind host plasminogen and mucin when exposed on the outer surface [29,30], while EF-Tu (tuf) and triosephosphate isomerase (tpiA) mediate direct interactions with host cell epithelial receptors, supporting robust in vivo intestinal colonization [42].

### 4.3. Biosynthesis and Evolutionary Origins of Postbiotic 5-MTP

A key finding of this study is the presence of the 5-MTP biosynthesis pathway in *L. paracasei* RM081, consisting of an antibiotic biosynthesis monooxygenase and a SAM-dependent methyltransferase. This dual-gene arrangement perfectly mirrors the canonical two-step biosynthesis pathway of 5-MTP observed in other systems (such as mammals), which requires an initial hydroxylation of tryptophan followed by a SAM-dependent O-methylation [14,43]. In bacteria, members of the antibiotic biosynthesis monooxygenase family and SAM-dependent methyltransferases are well-documented as tailoring enzymes capable of modifying aromatic rings and indole derivatives [44,45]. As an anti-inflammatory postbiotic metabolite endogenous to mammals, 5-MTP plays a critical physiological role in downregulating pro-inflammatory NF-κB pathways, inhibiting COX-2 expression, and protecting mucosal and endothelial barriers [43,46,47]. In a previous study, *L. paracasei RM081* was shown to produce 5-MTP, which decreased pro-inflammatory cytokines in macrophages and ameliorated DSS-induced colitis in mice [13].

Cross-species genomic screening revealed that the 5-MTP biosynthesis genes are not ubiquitous in LAB, but are highly conserved within the *L. casei* group and absent in other probiotic species (e.g., *L. plantarum*, *L. acidophilus*, and *Bifidobacterium*). This restricted distribution suggests a unique evolutionary niche. Flanking gene analysis showed that both the monooxygenase and methyltransferase genes are located on the chromosome in stable loci flanked by housekeeping metabolic genes (ABC transporter, threonine exporter), completely lacking insertion sequences (IS), transposases, or integrases in their immediate vicinity. Furthermore, phylogenetic analysis of the 5-MTP monooxygenase and methyltransferase sequences demonstrates that they cluster in a vertical topology that matches the standard species tree of *L. paracasei* and *L. casei*. This congruent phylogeny and stable genomic context indicate that the 5-MTP biosynthetic pathway is an ancestral/native trait within the *L. casei* group lineage rather than a recently acquired horizontal gene transfer (HGT) event.

The unique genomic capacity of *L. paracasei* RM081 to mediate 5-MTP biosynthesis implies that its anti-inflammatory mechanism provides a distinct functional niche, differentiating it from traditional probiotic strains that rely primarily on short-chain fatty acid (SCFA) production. Consequently, these insights highlight the profound clinical and industrial potential of *L. paracasei* RM081 as a uniquely qualified, postbiotic-driven therapeutic candidate tailored for mitigating intestinal inflammation and restoring mucosal homeostasis [13].

### 4.4. Safety Assessment and Non-Transferability

The safety of *L. paracasei* RM081 was validated through both genotypic and phenotypic assays. According to EFSA and FAO/WHO guidelines, probiotic strains must not carry transferable antimicrobial resistance determinants [48]. The strain contains no acquired, transferable AMR genes. Although phenotypic resistance to streptomycin and intermediate resistance to novobiocin were observed, genome analysis confirmed these are intrinsic, non-transferable traits. Streptomycin resistance is an intrinsic feature in *Lactobacillus* species, stemming from a lack of cytochrome-mediated active transport that fundamentally limits aminoglycoside uptake, compounded by a highly conserved configuration of the ribosomal target encoded by the *rpsL* gene [49,50]. Novobiocin intermediate resistance is directly attributable to the intrinsic structural characteristics of the DNA gyrase subunits (*gyrA* and *gyrB*), which serve as the native targets of the antibiotic without involving acquired mutational resistance [51]. Crucially, all these resistance-associated and virulence-niche determinants are strictly localized on the chromosome and are completely devoid of flanking mobile genetic elements such as insertion sequences, transposons, or integrons, which are key molecular hallmarks for confirming non-transferability [52,53]. The total absence of acquired, transferable resistance genes on plasmids or genomic islands definitively confirms that L. paracasei RM081 poses no risk of HGT to the host gut microbiome [54], ensuring its long-term safety for consumer health.

Furthermore, while traditional pathogens utilize virulence factors for infection, many of these genes are well-documented in probiotic lactic acid bacteria as beneficial factors for gastrointestinal survival and host interaction [55,56,57]. Our analysis confirmed the complete absence of true pathogenic toxins, such as functional enterotoxins or active cytotoxins. However, several genes flagged by databases as “virulence factors” actually function as essential colonization and stress-resistance mediators in probiotics. These included putative adhesins (*lap* and *efaA*) and moonlighting housekeeping genes (*eno* and *gapA*), which strongly corroborate the high Caco-2 adhesion rate observed in vitro [27,28,29,30]. Genes related to polysaccharide capsule synthesis (*cps*) and two-component stress response systems (*lisR*) were also detected, providing a genomic basis for the strain’s robust tolerance to acidic pH and bile salts [31,32]. Although a distant homolog of a hemolysin transporter was flagged by stringent database criteria, such occurrences are native, non-pathogenic membrane proteins commonly found in LAB genomes and lack true hemolytic functionality. Additionally, no genes associated with the production of biogenic amines (BAs), such as histidine decarboxylase, tyrosine decarboxylase, ornithine decarboxylase, lysine decarboxylase, and agmatine deiminase, were present. Collectively, these genomic findings confirm the absence of detrimental virulence determinants and toxic pathways, highlighting a genetic repertoire highly optimized for intestinal colonization and stress adaptation rather than pathogenesis, further robustly supporting the overall safety of *L. paracasei* RM081.

### 4.5. Methodological Limitations and Future Perspectives

While this study combines whole-genome sequencing with in vitro phenotypic assays to characterize the safety and probiotic potential of *L. paracasei* RM081, certain limitations must be addressed. WGS predicts the genetic potential and presence of metabolic pathways (such as stress tolerance, adhesion, and 5-MTP synthesis), but it does not confirm the active transcription or translation of these genes under physiological conditions.

To validate the functional expression of these predicted pathways during gastrointestinal transit and intestinal colonization, future studies employing transcriptomic (RNA-Seq) or RT-qPCR approaches are required. Analyzing the transcriptome of *L. paracasei* RM081 in response to gastric acid, bile salts, and co-culture with intestinal epithelial cells will confirm whether stress-survival genes (such as the F_0_F_1_ ATPase and efflux pumps), adhesins (such as *lap*, sortases, and moonlighting proteins), and the 5-MTP biosynthesis machinery are actively transcribed and functional in vivo.

## 5. Conclusions

In this study, a combination approach integrating genomic analysis and in vitro tests was used to evaluate the probiotic characteristics and safety of *L. paracasei* RM081, a strain isolated from raw bovine milk. The results demonstrated that *L. paracasei* RM081 exhibits strong resilience against gastrointestinal stressors, including acidic pH and bile salts, and possesses notable extracellular antioxidant capacity. In addition, the strain’s pronounced auto-aggregation, elevated cell surface hydrophobicity, and significant adherence to Caco-2 intestinal epithelial cells confirmed its robust capacity for intestinal colonization. Furthermore, our findings based on whole-genome sequence analysis revealed a safe genomic architecture. The *L. paracasei* RM081 genome harbors an extensive repertoire of carbohydrate-active enzymes, suggesting metabolic versatility for utilizing diverse prebiotics. Notably, genomic mining identified key determinants supporting its postbiotic capabilities, particularly the genetic potential for synthesizing the anti-inflammatory metabolite 5-MTP. Moreover, the *L. paracasei* RM081 genome lacks transferable antimicrobial resistance genes, virulence factors, and toxin-related genes. Overall, *L. paracasei* RM081 is considered safe and possesses the potential to exert beneficial probiotic and postbiotic properties. Further studies could be conducted to elucidate the application potential of *L. paracasei* RM081 in functional foods and targeted therapies aimed at enhancing gut homeostasis and immune regulation.

## Figures and Tables

**Table 1:** General genomic characteristics of *Lacticaseibacillus paracasei* RM081.

Feature	Value
Chromosome Size (bp)	3,084,987
Chromosome GC Content (%)	46.26%
Plasmid 1 Size (bp)	6696
Plasmid 2 Size (bp)	9600
Plasmid 3 Size (bp)	6373
Plasmid 4 Size (bp)	47,991
Total Genome Size (bp)	3,155,647
Average GC Content (%)	46.19%
Protein-coding genes (CDSs)	3096
tRNA genes	59
rRNA genes	15 (5 operons)
CheckM Completeness (%)	99.09%
CheckM Contamination (%)	0.74%
Deposition Accession (NCBI)	accession

**Table 2:** Carbohydrate fermentation profile of *Lacticaseibacillus paracasei* RM081 analyzed by API 50 CH.

Carbohydrate	Result	Carbohydrate	Result	Carbohydrate	Result
Glycerol	-	D-mannitol	+	D-raffinose	+
Erythritol	-	D-sorbitol	-	Starch	-
D-arabinose	-	Methyl-α-D-mannopyranoside	-	Glycogen	-
L-arabinose	+	Methyl-α-D-glucopyranoside	-	Xylitol	-
D-ribose	+	N-acetylglucosamine	+	Gentiobiose	+
D-xylose	-	Amygdalin	-	D-turanose	+
L-xylose	-	Arbutin	+	D-lyxose	-
D-adonitol	-	Esculin ferric citrate	-	D-tagatose	+
Methyl-β-D-xylopyranoside	-	Salicin	+	D-fucose	-
D-galactose	+	D-cellobiose	+	L-fucose	-
D-glucose	+	D-maltose	+	D-arabitol	-
D-fructose	+	D-lactose	+	L-arabitol	-
D-mannose	+	D-melibiose	-	Potassium gluconate	-
L-sorbose	-	D-sucrose	+	Potassium 2-ketogluconate	-
L-rhamnose	-	D-trehalose	+	Potassium 5-ketogluconate	-
Dulcitol	-	Inulin	-		
Inositol	-	D-melezitose	+		

Note: (+), positive reaction; (-), negative reaction.

**Table 3:** Cell surface properties of *Lacticaseibacillus paracasei* RM081.

Cell Surface Properties	Value	Interpretation
Auto-aggregation (5 h)	85.0 ± 0.7%	High auto-aggregation
Cell surface hydrophobicity (n-hexadecane)	71.5 ± 2.4%	High hydrophobic

**Table 4:** Antibiotic susceptibility profile of *Lacticaseibacillus paracasei* RM081.

Antibiotic Susceptibility	Concentration per Disc (μg per Tablet)	Diameter (mm)	Interpretation
Penicillin	10	35.0 ± 0.5 mm	Susceptible
Erythromycin	15	32.0 ± 0.6 mm	Susceptible
Chloramphenicol	30	30.0 ± 0.3 mm	Susceptible
Tetracycline	30	30.0 ± 0.3 mm	Susceptible
Streptomycin	10	0 mm	Resistant
Novobiocin	5	20.2 ± 0.4 mm	Intermediate

Antibiotic susceptibility was evaluated according to the diameter of the inhibition zone: resistant (≤14 mm); intermediate (15–19 mm); susceptible (≥20 mm). The results were interpreted based on the CLSI criteria.

**Table 5:** In silico prediction of safety-related genomic features in *Lacticaseibacillus paracasei* RM081.

Feature Category	Detected Elements/Status	Functional Implication
Antimicrobial Resistance (AMR)	None detected	Indicates safe genomic profile for probiotic use
Biogenic Amine (BA) Production	None detected	Safe for human consumption
Plasmids	4 plasmids (No AMR genes)	Safe; no mobile AMR elements detected
Prophages	5 intact regions (No AMR genes)	Safe; no mobile AMR elements detected
Virulence Factors	*lap*, *efaA*	Putative adhesins; beneficial for gut colonization [27,28]
	*eno*, *gapA*	Moonlighting proteins; support intestinal adhesion [29,30]
	*cps*	Polysaccharide capsule synthesis; aids in stress tolerance [31]
	*lisR*	Two-component systems; enhance acid and bile survival [32]
	Hemolysin transporter homolog	Common in LAB; non-pathogenic trait [33]

## Data Availability

The raw sequencing reads (Illumina and PacBio) and the assembled genome of *L. paracasei* RM081 have been submitted to the NCBI BioProject database under BioProject accession number PRJNA1483404. The raw data supporting the conclusions of this article will be made available by the authors on request, and the sequence data will be publicly released upon publication.

## Supplementary Materials

The following supporting information can be downloaded at: https://www.mdpi.com/article/10.3390/microorganisms14071431/s1, Table S1: General genomic characteristics of the 20 Representative *Lacticaseibacillus paracasei* genomes used for comparative genomics; Figure S1: Functional annotation of the *Lacticaseibacillus paracasei* RM081 genome; Figure S2: Five-methoxytryptophan (5-MTP) biosynthesis genes phylogenetic tree. Evolutionary relationships of the 5-MTP biosynthesis genes (antibiotic biosynthesis monooxygenase and SAM-dependent methyltransferase) among the *Lacticaseibacillus casei* group and related taxa. The vertical inheritance topology closely mirrors the standard taxonomic species tree, indicating that these biosynthetic genes are ancestral and native traits within the L. casei group lineage, rather than having been acquired via horizontal gene transfer (HGT).
