# Supplementary material for: Whole-Genome Sequence Analysis and Probiotic Characterization of 5-Methoxytryptophan-Producing Strain Lacticaseibacillus paracasei RM081
Source: Microorganisms. 2026 Jun 30;14(7):1431. doi: 10.3390/microorganisms14071431 (PMC13413441; doi:10.3390/microorganisms14071431)
Supplement: Supplementary file 1 [file microorganisms-14-01431-s001.zip › microorganisms-4362383-supplementary.pdf]

Microorganisms Supplementary materials for:

Whole-genome sequence analysis and probiotic characterization of 5-methoxytryptophan-producing strain *Lacticaseibacillus paracasei* RM081

Yu-Yi Chen[12], Alican Abay[12], Muhammet Ali ASAN[12], Yu-Chun Lin[3*] and Yen-Po Chen [12*]

[1] Department of Animal Science, National Chung Hsing University, Taichung City 402, Taiwan;
[2] The iEGG and Animal Biotechnology Research Center, National Chung Hsing University, Taichung City 402, Taiwan
[3] Department of Biotechnology and Animal Science, National Ilan University, Yilan County 260, Taiwan;
* Correspondence: chenyp@nchu.edu.tw

Table S1. General genomic characteristics of 20 representative *Lacticaseibacillus paracasei* strains used for comparative genomics

**Strain**	**Accession**	**Genome Size (Mb)**	**GC Content (%)**	**Completeness (%)**	**Contamination (%)**	**Isolation Source / Host**
L. paracasei 347-16	GCF_012955485.1	3.22	46%	94.59%	7.56%	feces
L. paracasei ATG-E1	GCF_026013725.1	3.22	46.5%	90.51%	10.49%	Homo sapiens
L. paracasei BBM398	GCF_046581425.1	3.01	46.5%	92.60%	3.65%	the feces of healthy humans
L. paracasei CLP-C10	GCF_028609725.1	3.23	46%	96.45%	11.70%	fermented milk
L. paracasei CLP-Y5	GCF_028609745.1	3.18	46.5%	93.42%	11.70%	fermented milk
L. paracasei CUDS0725	GCF_037414395.1	3.04	46.5%	91.82%	4.13%	feces
L. paracasei D11	GCF_056725945.1	3.04	46.5%	92.52%	3.35%	soymilk fermentation
L. paracasei HD1.7	GCF_002865565.1	3.04	46.5%	87.77%	8.13%	Chinese sauerkraut
L. paracasei HP-B1337	GCF_035586595.1	3.10	46.5%	93.76%	5.62%	Environment/Other
L. paracasei L14	GCF_030480425.1	3.00	46.5%	90.09%	3.56%	raw milk
L. paracasei LC2W	GCF_000194785.1	3.08	46.5%	91.82%	5.69%	traditional dairy products
L. paracasei Lpc10	GCF_003199005.1	3.05	46.5%	95.32%	7.81%	Patagonian Merlot Wine
L. paracasei M8	GCF_051123965.1	3.29	46.5%	95.99%	2.49%	fermented tofu whey
L. paracasei NCU215	GCF_052907405.1	3.15	46.5%	94.65%	6.51%	fermented vegetable
L. paracasei ORD 0998	GCF_041283745.2	3.15	46.5%	94.65%	6.51%	vaginal specimen from healthy woman
L. paracasei SMN-LBK	GCF_024498315.1	3.15	46.5%	94.65%	6.51%	koumiss
L. paracasei TCI727	GCF_040267725.1	3.10	46.5%	93.00%	5.11%	raw milk
L. paracasei TK-P4A	GCF_015377585.1	3.08	46.5%	93.17%	7.87%	probiotic products
L. paracasei VHProbi O44	GCF_025252385.1	3.32	46%	92.95%	9.69%	dairy product
L. paracasei YGRT25	GCF_041734755.1	3.10	46.5%	91.39%	6.16%	yogurt

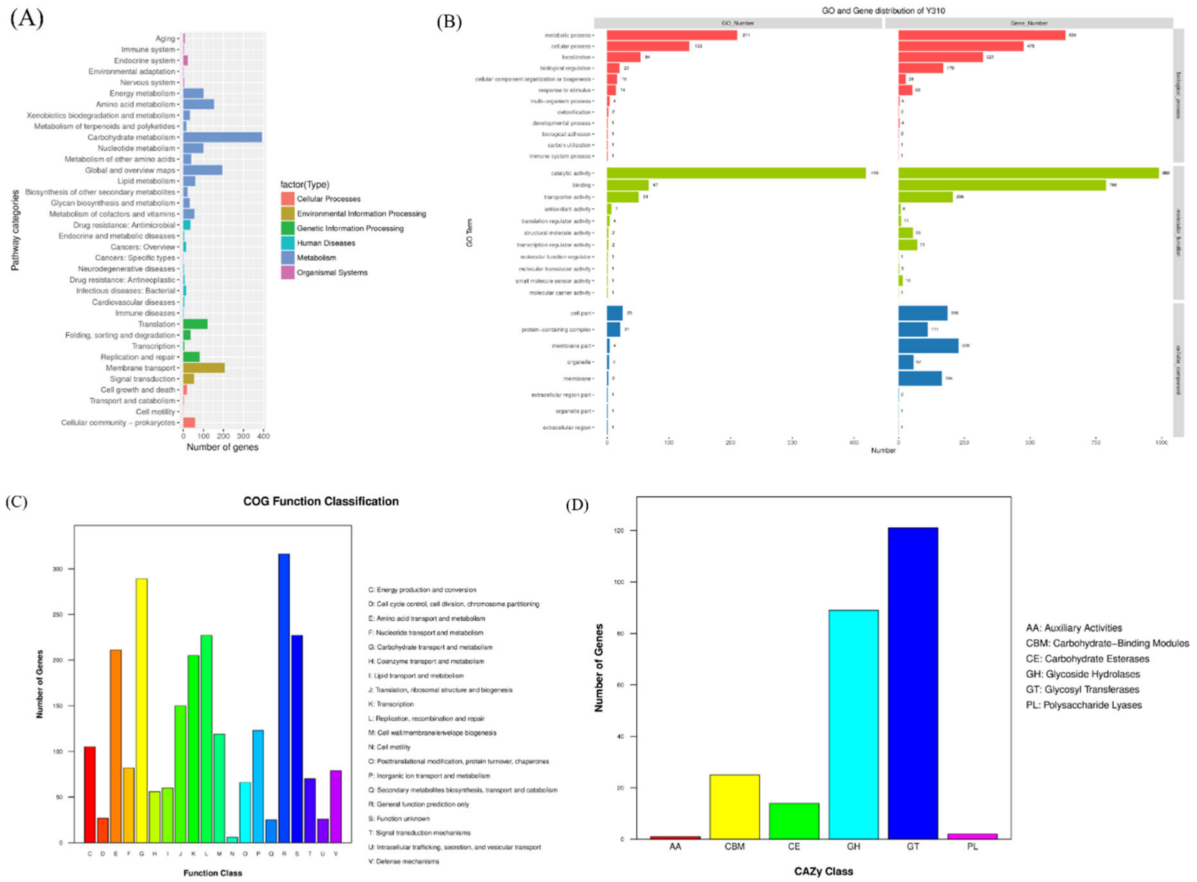

Figure S1. Functional annotation of the ***Lacticaseibacillus paracasei*** **RM081 genome.** Distribution of predicted genes assigned to (A) Kyoto Encyclopedia of Genes and Genomes (KEGG) pathways, (B) Gene Ontology (GO) terms, (C) Clusters of Orthologous Groups (COG) functional categories, and (D) Carbohydrate-Active enZYmes (CAZy) classes.

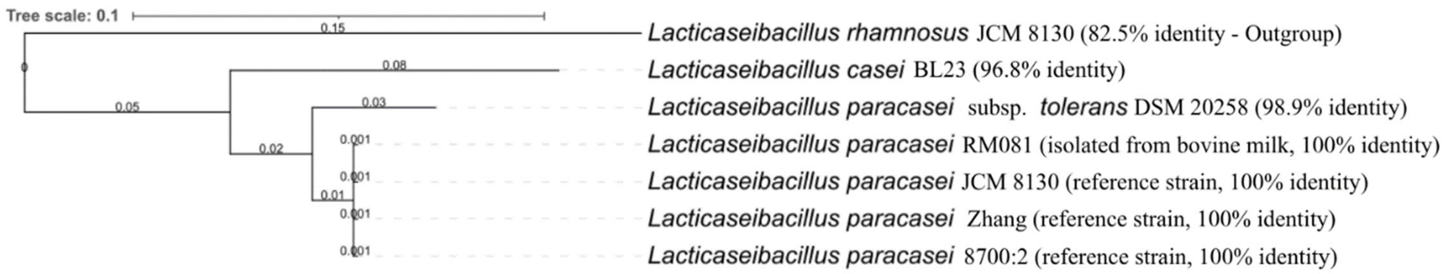

Figure S2. Five-methoxytryptophan (5-MTP) biosynthesis genes phylogenetic tree. Evolutionary relationships of the 5-MTP biosynthesis genes (antibiotic biosynthesis monooxygenase and SAM-dependent methyltransferase) among the *Lacticaseibacillus casei* group and related taxa. The vertical inheritance topology closely mirrors the standard taxonomic species tree, indicating that these biosynthetic genes are ancestral and native traits within the *L. casei* group lineage, rather than having been acquired via horizontal gene transfer (HGT).
